# Minimizing Injury and Maximizing Return to Play: Lessons from Engineered Ligaments

**DOI:** 10.1007/s40279-017-0719-x

**Published:** 2017-03-22

**Authors:** Keith Baar

**Affiliations:** 1 Department of Neurobiology, Physiology and Behavior, University of California Davis, One Shields Ave, Davis, CA 95616 USA; 2 Department of Physiology and Membrane Biology, University of California Davis, One Shields Ave, Davis, CA 95616 USA

## Abstract

Musculoskeletal injuries account for more than 70% of time away from sports. One of the reasons for the high number of injuries and long return to play is that we have only a very basic understanding of how our training alters tendon and ligament (sinew) structure and function. Sinews are highly dense tissues that are difficult to characterize both in vivo and in vitro. Recently, engineered ligaments have been developed in vitro using cells from human anterior cruciate ligaments or hamstring tendons. These three-dimensional tissues can be grown in a laboratory, treated with agents thought to affect sinew physiology, and then mechanically tested to determine their function. Using these tissues, we have learned that sinews, like bone, quickly become refractory to an exercise stimulus, suggesting that short (<10 min) periods of activity with relatively long (6 h) periods of rest are best to train these tissues. The engineered sinews have also shown how estrogen decreases sinew function and that a factor released following intense exercise increases sinew collagen synthesis and function. Last, engineered sinews are being used to screen possible nutritional interventions that may benefit tendon or ligament function. Using the data derived from these tissue-engineered sinews, new nutritional and training regimes are being designed and tested with the goal of minimizing injury and accelerating return to play.

## Incidence of Soft-Tissue Injury in Sport

Soft-tissue injuries, those affecting muscles, tendons, and ligaments, are extremely common at all levels of sport. In youth sport, ~50% of all injuries are sprains [1]. In college athletics, the rates of ankle sprains remained relatively constant, at ~1 per 1000 athlete exposures, over the 15 years between 1988 and 2004 [2]. By contrast, anterior cruciate ligament (ACL) ruptures increased by 1.3% per year to 0.14 per 1000 athlete exposures over the same time period. In professional sports, the incidence of soft-tissue injury for players reaches 60% for the English Premier League [3] and nearly 70% in the National Football League [4]. Beyond sporting populations, diabetic individuals are up to ten times more likely to experience tendon injuries than non-diabetic individuals [5], and tendon injuries increase with aging as evidenced by the fact that 80% of people in their 80 s have experienced a ruptured tendon [6]. Even though musculoskeletal injuries are extremely common and have huge personal, competitive, and financial costs, very few advances have been made in preventing these injuries.

**Fig. 1:**
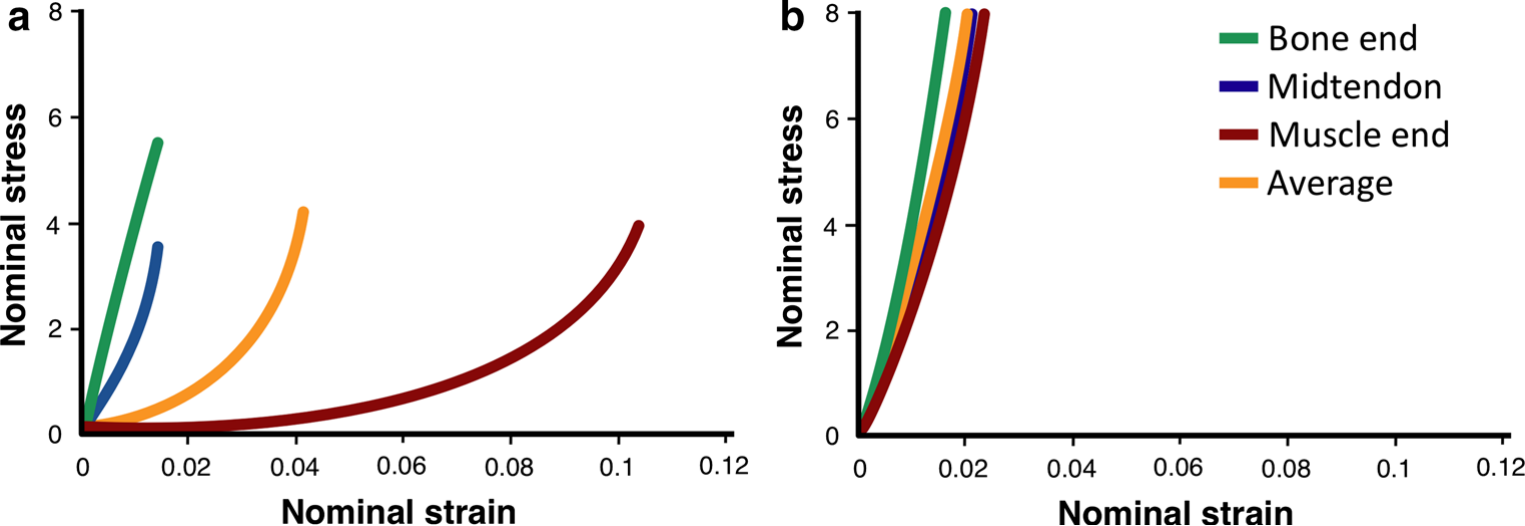
Regional mechanics of tendon. The mechanics of **a** healthy tendon or **b** tendon after 5 weeks of immobilization. Note that in a healthy tendon, the muscle end of the tendon (*red*) stretches much more than the bone end (*green*), whereas the mid-tendon region shows intermediate mechanics. By contrast, after forced inactivity, all regions of the tendon become stiffer Adapted from Arruda et al. [7]

Tendons and ligaments are often grouped together into a single category (termed sinew in this review) because of similarities in their molecular composition, structure, and general function. However, the importance of the fundamental difference, that a tendon attaches a compliant tissue to a stiff tissue, whereas a ligament attaches two stiff tissues, is often underappreciated. The connection of two mechanically disparate tissues could result in stress and strain concentrations (i.e., one tissue stretches much more than another at the interface), which would lead to failure (rupture). The tendon overcomes this issue by being a variable mechanical tissue (Fig. 1) [7]. This means that a healthy tendon is more compliant near the muscle and becomes stiffer as it nears the bone. The compliant region of a tendon is believed to protect the attached muscle from injury, by acting as a shock absorber. For example, during running, a healthy tendon lengthens to absorb energy allowing the muscle to contract isometrically [8]. However, when tendon stiffness exceeds the isometric strength of the muscle, either the biomechanics of the movement must change or the muscle is forced to undergo potentially damaging lengthening contractions. Functionally, this means that even though in both tendons and ligaments ultimate tensile strength (load at which they fail) increases as a function of stiffness [9], a stiffer tendon is more likely to produce muscle damage during the same exercise than a healthy compliant tissue [10].

The varying mechanics within a healthy tendon are achieved by variations in the orientation and crosslinking of the collagen within the tendon. Along the length of a tendon, the molecular crosslinks that connect adjacent collagen molecules and fibrils increase [11], and crosslinking is known to increase the stiffness of a tendon [12]. Therefore, because there are more crosslinks near the bone than the muscle, stiffness increases moving from muscle to bone [7, 13]. Interestingly, when a joint is inactive (such as when in a cast) or the muscle is prevented from contracting against load, the tendons that cross that joint lose the compliant region and become stiffer close to the muscle [7, 13], likely because of an increase in crosslinks.

From the background above, the most important messages are: (1) tendon/ligament injuries are common in daily activities and at every level of athletic performance, (2) a healthy tendon has varying stiffness along its length and the compliant region acts as a shock absorber that protects the muscle from injury, and (3) as a result of the tissues they attach, tendon and ligament are functionally different: the stiffer the ligament the better, whereas if tendon stiffness is too high injuries will increase to the associated muscles.

## Modeling Sinew Physiology In Vitro

**Fig. 2:**
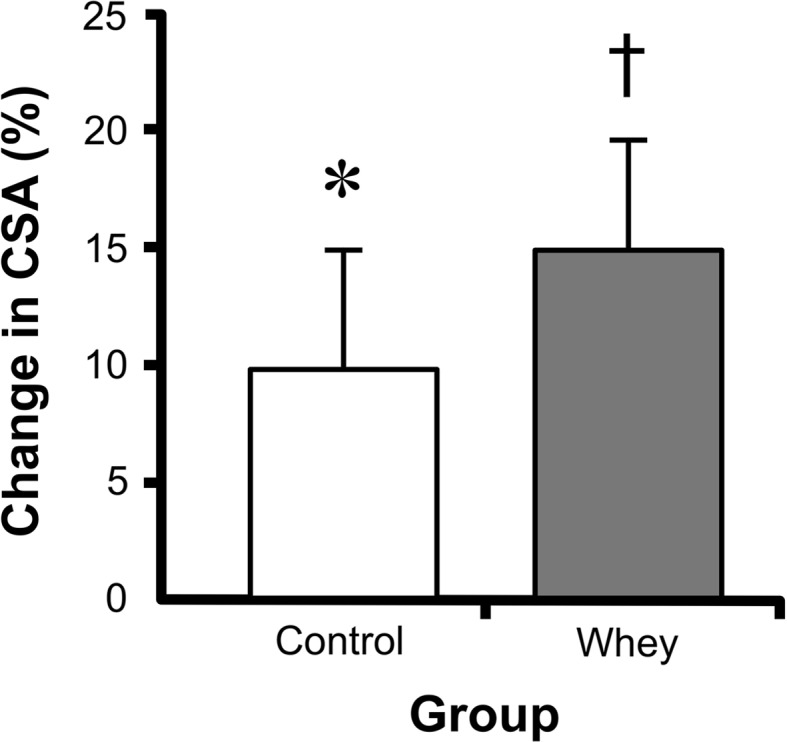
Change in patellar tendon cross-sectional area (CSA) with training and nutrition. Twelve weeks of strength training resulted in a 10% increase in the tendon CSA in the placebo control group and a 15% hypertrophy in the group that performed the resistance exercise training and supplemented their training with 19.5 g of whey protein. *Significantly different than pre-training (*p* < 0.05); ^†^ significantly different from pre-training (*p* < 0.001). Data are means ± standard error of the mean Adapted from Farup et al. [15]

**Fig. 3:**
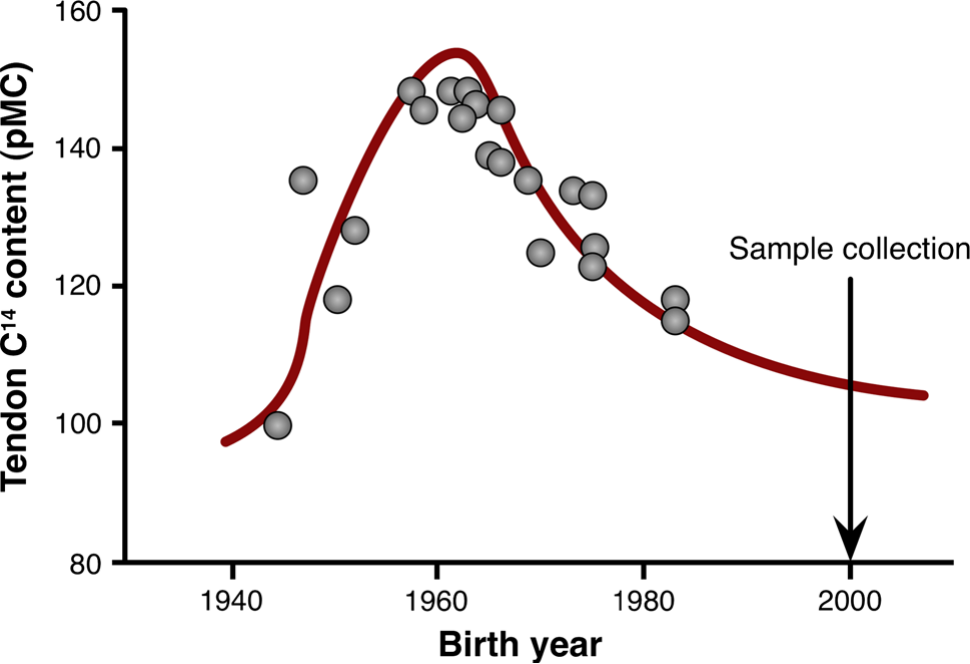
Slow turnover of collagen within the central core of the Achilles tendon. The amount of C^14^ as a percent modern carbon (pMC) in collagen isolated from the central core of the Achilles tendon was compared with that in the atmosphere over time to determine the rate of collagen turnover in the Achilles tendon. The relationship between the C^14^ levels in the central core and that found in the atmosphere indicates that the turnover rate of collagen is extremely slow. In fact, most of the samples measured indicated that the collagen in the center of the Achilles had not turned over since the individual was 17 years of age Adapted from Heinemeier [17]

For years, scientists, coaches, and athletes considered tendons and ligaments mechanical bands that did not respond to exercise. However, it is now clear that these tissues respond to loading. For example, the patellar tendon in the dominant leg of fencers and badminton players is 20–30% larger than that of their trail leg [14]. Further, nutritional interventions, such as consuming a whey protein supplement, have the potential to increase tendon hypertrophy that results from strength training (Fig. 2) [15]. This means that sinews are dynamic tissues. Interestingly, the existing data suggest that tendons rapidly respond to changes in muscle strength, possibly to minimize changes in the peak strain across the tendon during contraction [16]. However, it is important to note that even though part of the tissue is dynamic, the collagen at the core of a tendon does not turn over between the ages of 17 and 70 years (Fig. 3) [17]. Together, these data suggest that an adult tendon grows like a tree, adding and removing collagen only on the outside [18].

Because tendons are dynamic tissues, understanding how exercise and nutrition increase the synthesis of collagen and improve tendon function could allow us to improve performance, prevent injury, and accelerate return to play. However, unlike many tissues, tendons largely comprise extracellular proteins. In fact, the number of cells within a tendon decreases with age until in adult tendons, fewer than 0.01 cells/µm^2^ remain [19]. Therefore, obtaining sufficient intracellular protein from a tendon biopsy to perform standard biochemical assays can be extremely challenging. Together with the fact that proteins within the core of the tendon turn over very slowly [17], this means that it is very difficult to understand how the cells within a healthy adult tendon respond to nutrition or exercise.

**Fig. 4:**
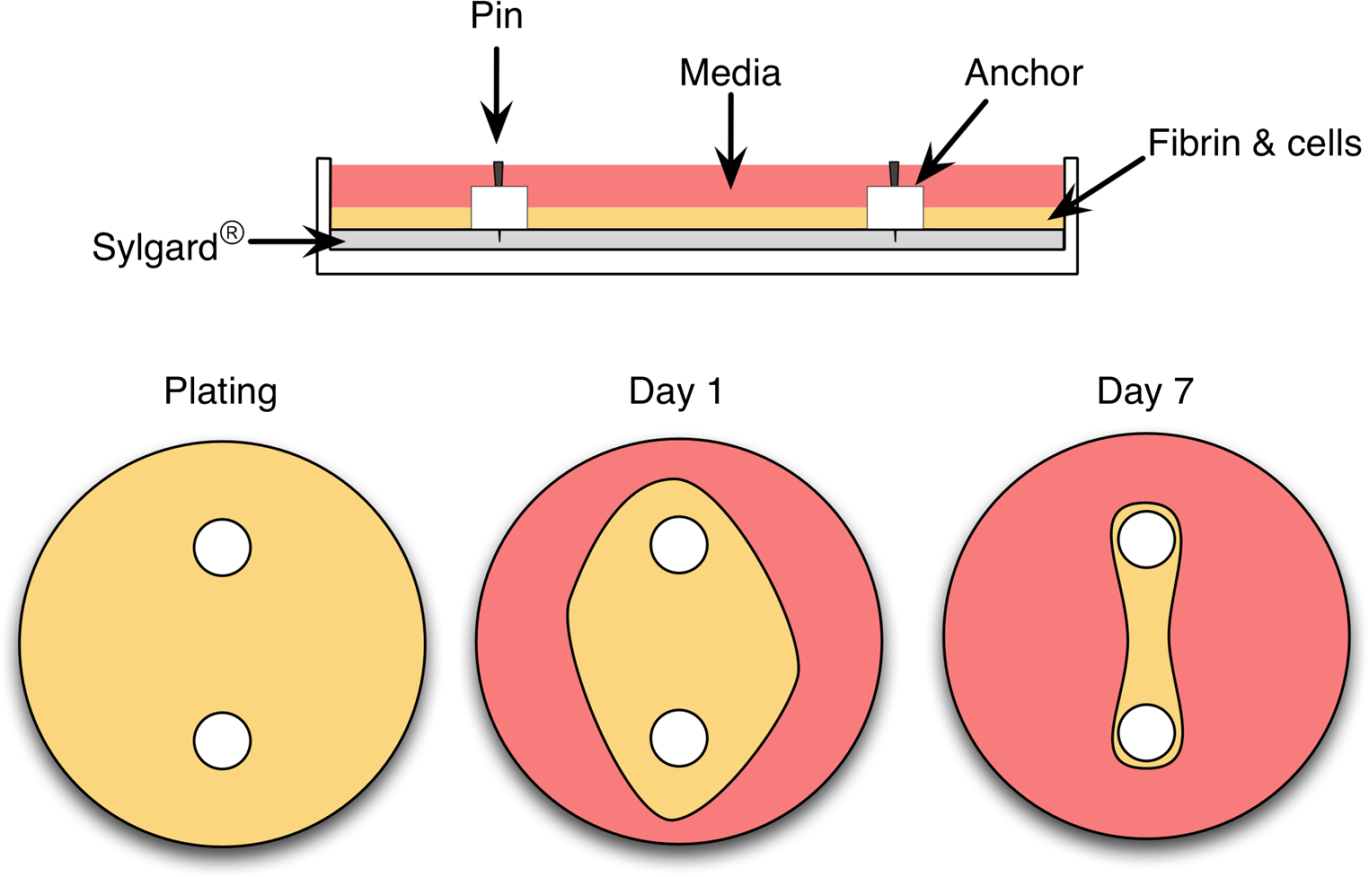
Engineered ligament model. Engineered ligaments can be formed by embedding human anterior cruciate ligament fibroblasts into a fibrin gel. A tubular ligament results from limiting the natural contraction of the gel using anchors pinned into a tissue culture plate that has been modified such that the bottom of the plate is coated with a silicone polymer (polydimethylsiloxane; Sylgard^®^). Following plating, the cells within the fibrin gel (depicted in *yellow*) contract the gel around the anchors forming a tubular ligament by day 7 in culture

For these reasons, over the last 10 years, my laboratory and others have developed a three-dimensional model of a human tendon/ligament [20–24]. To achieve this goal, we isolate human fibroblasts from the remnants of ruptured ACLs collected during reconstructive surgery [25]. The collagen within the ACL is enzymatically digested and the cells from within this matrix are released and grown in culture. The cells can be expanded and this allows us to make hundreds of ligaments from the same donor, eliminating any genetic differences between subjects. To engineer ligaments, cells are embedded into a gel made from fibrin, the same protein that forms blood clots. We use this matrix because it is the biological matrix that the cells are exposed to in development or during injury repair [26]. Furthermore, using fibrin instead of collagen allows us to quantify collagen production as an outcome measure in our experiments because any collagen in the graft has to have been made by the cells. The cells continue to divide within the fibrin gel and over 7 days contract around two calcium phosphate cement anchors that are placed in the culture dish to serve as bones (Fig. 4). After 7 days, the fibrin contracts into a single tissue between the anchors and continues to develop like an embryonic tendon or ligament [21]. As with developing tendons/ligaments [27], these engineered tissues have more cells and less matrix [21], their rate of collagen synthesis is significantly higher [28], they express more developmental collagen isoforms [20], and they are much weaker than adult sinews [24]. Despite these significant differences, these engineered tissues may provide a model that will be useful in understanding the effects of exercise and nutrition on tendon/ligament function. Subsequent sections outline some of the exciting findings using this model, how these data compare with the animal/human data (where possible), and how what we are learning using this model can improve performance, decrease injuries, and accelerate return to play.

## Loading and Sinew Function

Tendons, like other forms of fibrous connective tissue such as bone and ligaments, adapt to their loading state. In the tendons of mature animals and humans, disuse leads to a decrease in total collagen [29, 30], whereas acute exercise increases the rate of collagen deposition [31]. Even though activity is known to regulate cellular processes within tendons and ligaments, the effect of different frequencies, intensities, and durations of exercise is largely unstudied. To begin to understand what type of activity was optimal for sinew, engineered ligaments were stretched at different frequencies, intensities, and durations and the molecular response was determined [25]. These experiments showed that the molecular response to loading was independent of the frequency and intensity of loading, because ranges from 1 load/10 s to 1 load/s and from 2.5 to 10% stretch produced the same molecular response. This is consistent with animal experiments where the molecular response to resistance exercise was the same in tendons regardless of whether the muscle underwent shortening, isometric, or lengthening contractions [31]. The only parameter that did alter the cellular response was time. Within 10 min of starting the activity, the molecular response had reached its maximum. If loading continued, the molecular signals began switching off [25]. Further experiments showed that it took 6 h for the cells to become responsive to exercise again [25]. Using this information, we developed an intermittent exercise program consisting of 10 min of activity followed by 6 h of rest. After 5 days, the engineered ligaments that had undergone the intermittent activity protocol produced more collagen than those that were exercised continuously [25]. These results are similar to what we know occurs in bone in vivo; very few loading events followed by 6–8 h of rest result in the greatest amount of bone mineral deposition [32].

Clinically, these data suggest that limited range-of-motion exercises even if performed with a light weight should be effective at increasing collagen synthesis in a developing or regenerating tendon or ligament. From our model and the existing in-vivo data, the important message is that: repeated short periods of activity that load the connective tissue followed by long periods of rest appear to be optimum for connective tissue health and function.

## Hormonal Effects on Sinew Function

Female athletes participating in cutting and jumping sports have a four to six times greater incidence of ACL rupture than their male counterparts [33]. Interestingly, the degree of knee laxity [34, 35] and incidence of ACL rupture [36] are related to circulating estrogen levels. This suggests that even though there are well-established biomechanical differences between men and women, hormone levels might directly affect ligament function. To attempt to understand the mechanism underlying this effect, we mimicked the estrogen surge that occurs just before ovulation by transiently increasing estrogen levels in our culture model and measuring the resulting changes in mechanics [37]. Interestingly, as little as 48 h in physiologically high estrogen was enough to decrease the stiffness of our ligaments without any change in the collagen content. A change in stiffness without a concomitant change in collagen suggested that there was a change in the crosslinking of the collagen. To directly test this hypothesis, we treated our ligaments with high estrogen for 24 or 48 h and measured the activity of lysyl oxidase, the primary collagen crosslinking enzyme in ligaments. Consistent with our hypothesis, estrogen decreased the activity of lysyl oxidase by more than 80% at the 48-h timepoint even though its expression only decreased by 20% [37]. This indicates that the transient increase in estrogen during the menstrual cycle decreases enzymatic crosslinking, resulting in a decrease in stiffness that places the ACL at a greater risk of failure.

While estrogen is known to decrease sinew function, as described in Sect. 3, exercise has a positive effect. While most of the benefit of exercise is the result of the direct effect of loading [15, 25], exercise is known to have a global positive effect on collagenous tissues [38]. To test whether the hormonal changes that result from strength training are beneficial for sinew function, our team drew blood from 12 healthy young male individuals before and after resistance exercise [39]. The serum from these subjects before and after exercise was isolated and then used in the growth media of the engineered ligaments. The effect of the different sera on collagen synthesis and mechanics was then determined. Constructs grown in the post-exercise serum showed a significant increase in collagen content and mechanics, suggesting that something within the exercised serum improves sinew function [39]. The results showed that the effect was not mediated by growth hormone or insulin-like growth factor-1, but that the hormone did signal through the phosphoinositol 3-kinase/mechanistic target of rapamycin complex 1 (mTORC1) and extracellular regulated kinase pathways. These data suggest that exercise produces a global signal that improves connective tissue function. Identifying this factor might provide a mechanism to improve sinew health and function, and accelerate return to play.

Another significant finding arising from the study of the effects of serum on engineered ligament function was that treatment with high levels of recombinant growth hormone had no effect on the collagen content or mechanics of our grafts [39]. In contrast, insulin-like growth factor-1 increased engineered ligament collagen and mechanics in a dose-dependent fashion. These data support the idea that within the physiological range, growth hormone has little direct effect on sinew function [40]. However, growth hormone can increase collagen synthesis and improve recovery following immobilization, likely through its regulation of insulin-like growth factor-1 [41, 42].

## Nutritional Interventions to Improve Soft-Tissue Function

Compared with muscle, the science of nutritional interventions that can improve soft-tissue function in humans is in its infancy. One article has shown that whey protein can improve tendon hypertrophy in response to strength training [15]. However, whether this was the result of a direct effect on the tendon or an indirect effect related to greater muscle hypertrophy and strength gains is still unclear. In support of a role for whey, a similar tendon hypertrophy was seen in rats following 5 weeks of leucine supplementation after a period of malnutrition [43]. The molecular target of whey and leucine is mTORC1 [44], and as described in Sect. 4, mTORC1 was also activated by post-exercise serum. Therefore, to determine whether mTORC1 activity was important in collagen synthesis and sinew mechanics, our team added rapamycin to the culture media to specifically block mTORC1. Seven days of treatment with rapamycin decreased the mechanics and collagen content of the grafts by more than 50% (unpublished observation). However, whether these data are the result of a decrease in collagen synthesis or a decrease in cell proliferation within our model has yet to be determined. Regardless, these data suggest that using leucine-rich whey protein can activate mTORC1 within sinews and increase collagen synthesis. However, the effects of whey protein on sinew structure and mechanics in humans have yet to be determined.

Other than leucine-rich protein, no nutritional interventions have been shown to have an effect on sinew in humans. However, using the tissue-engineered ligament model, our team has shown that amino acids that are enriched in collagen (proline, hydroxyproline, and hydroxylysine) added together with vitamin C can improve collagen synthesis [24]. The vitamin C effect is not surprising given that vitamin C deficiency results in scurvy, a disease characterized by a loss of collagen [45]. Vitamin C functions within connective tissues as an essential co-factor for prolyl 4-hydroxylase, an enzyme required for hydroxylation of proline and the synthesis and secretion of procollagen [45]. The amino acids that have a positive effect on collagen synthesis in our model include glycine, proline, lysine, hydroxylysine, and hydroxyproline. These amino acids are the main components of collagen, suggesting that even in our in-vitro model, where amino acids are five times the physiological level, excess proline, lysine, and their hydroxylated analogs can still be beneficial. Interestingly, these same amino acids are enriched in gelatin, which is usually made from the skin, tendon and ligaments of cows or pigs. We have now begun feeding gelatin to people and have seen extremely positive responses on collagen production and return to play in athletes after injury. In fact, in a randomized, double-blind crossover clinical trial, we found that ingestion of 15 g of gelatin 1 h before 6 min of jump rope is able to double collagen synthesis [46]. Further, serum taken before or 1 h after feeding a placebo or 5 or 15 g of vitamin C-enriched gelatin results in a dose-dependent increase in collagen in engineered ligaments [46]. These data support the use of gelatin as a nutritional intervention to increase collagen synthesis in sinew and bone.

## Conclusions and Science-Based Recommendations for Training to Improve Tendon Health and Performance

From the background provided above, a series of recommendations can be developed to maximize performance, decrease the risk of tendon/ligament injury, and/or accelerate return to play.

- Consider incorporating a connective tissue health session into training. This type of session would involve <10 min of activity targeted to a tendon/ligament that is prone to injury. For example, runners would do a session to target the hamstrings and patellar and Achilles tendons, whereas baseball players would target the throwing arm. These exercises could be performed with a light weight and using a limited range of motion if necessary. The connective tissue health session should be performed either 6 h before or after any other training.
- Following injury, athletes should begin training as soon as possible. Training can consist of simple range-of-motion and limited weight supported exercises because the amplitude of the load is not important for stimulating collagen production [25]. The training should again consist of <10 min of activity followed by 6 h of rest. Reasonably, this means that the athlete will train for three short periods each day.
- Consume leucine-rich protein as part of training. Beyond the direct effects this will have on muscle [47], tendons will also benefit from the added muscle mass and strength and possibly a greater mTORC1 activation [15].
- Glucose uptake into tendons increases during exercise [48]. However, because blood flow to inactive tendons is limited, nutrient delivery to tendons following exercise is believed to be relatively low. This suggests that any nutritional intervention that is designed to directly target a specific tendon/ligament needs to be in place prior to exercise.
- Thirty to sixty minutes before training, athletes should be encouraged to consume 15 g of gelatin in either a liquid or gel form [46]. The exact amount of gelatin and whether this will vary with body weight is currently being determined.
